# Human bioimpedance-based state detection technologies for sports health monitoring: a review

**DOI:** 10.3389/fphys.2026.1911660

**Published:** 2026-09-04

**Authors:** Yuanqingqing Tao, Ziyi Hao, Guo Li

**Affiliations:** 1 School of Physical Education, Nanjing Tech University, Nanjing, China; 2 Faculty of Education, The Chinese University of Hong Kong, Hong Kong, Hong Kong SAR, China

**Keywords:** bioelectrical impedance analysis, human bioimpedance, multimodal monitoring, physiological state assessment, sports health monitoring, wearable sensing

## Abstract

With the increasing demand for personalized exercise guidance and real-time health assessment, sports health monitoring is shifting from single-index measurement toward continuous and objective human state assessment. Human bioimpedance, used in this review as an umbrella term for the complex electrical impedance measured in human tissues, has become an important physiological sensing approach owing to its portability, operational safety, and compatibility with wearable platforms. The measured impedance is decomposed into resistance and reactance, from which impedance magnitude and phase angle (PhA) are derived, whereas bioelectrical impedance analysis (BIA), bioimpedance spectroscopy (BIS), and electrical impedance myography (EIM) represent distinct analytical or regional assessment frameworks. These measurements can provide information related to body composition, fluid distribution, membrane-associated polarization, and local tissue status. This review summarizes recent advances in human bioimpedance for sports health monitoring, focusing on its physiological basis, impedance models, measurement principles, parameter interpretation, and applications in body composition, hydration, fatigue, muscle function, and multimodal monitoring. Existing studies indicate substantial potential for repeated and individualized assessment; however, practical use remains limited by measurement repeatability, motion artifacts, electrode–skin interface stability, model generalizability, and insufficient validation under dynamic conditions. Future research should strengthen standardized reporting, wearable acquisition, multisource data fusion, and physiologically grounded analytical methods.

## 1 Introduction

Sports health monitoring has become an important approach for understanding exercise-induced changes in human states. By providing continuous and objective information during physical activity, it helps identify adaptive responses and potential health risks. Regular physical activity has been widely recognized as an effective strategy for enhancing physical fitness, preventing chronic diseases, promoting functional recovery, and supporting healthy aging (Tong and Ye, 2023). However, physiological responses to exercise vary considerably among individuals and are influenced by factors such as training intensity, physical condition, recovery capacity, and health status. Excessive training loads, inadequate recovery, or inappropriate exercise interventions may result in fatigue accumulation, sports injuries, and adverse health outcomes. Consequently, scientific assessment and management of exercise-related physiological states have become increasingly important in sports medicine, rehabilitation, preventive healthcare, and personalized health management. With the rapid development of wearable sensing technologies and intelligent healthcare systems, sports health monitoring has emerged as a key research field for continuous physiological assessment and individualized health management (Ma et al., 2024). Compared with conventional approaches relying on intermittent measurements or subjective evaluations, modern sports health monitoring emphasizes the continuous acquisition and integrated interpretation of physiological signals, motor function indicators, and fatigue–recovery characteristics, thereby providing objective support for exercise load optimization, athletic performance evaluation, injury prevention, rehabilitation guidance, and personalized health interventions (Almetwally et al., 2025; Parollo et al., 2023).

Physiological state detection constitutes a fundamental component of sports health monitoring and directly affects the accuracy of exercise response assessment, fatigue evaluation, movement quality analysis, and health risk identification (Ji and Zhu, 2024). To date, electrocardiography (ECG), electromyography (EMG), electroencephalography (EEG), photoplethysmography (PPG), inertial sensing, electrodermal activity (EDA), and temperature sensing have become widely adopted approaches for monitoring physiological and behavioral states during exercise (Zhao et al., 2023; Butkevičiūtė et al., 2019). These technologies provide valuable information regarding cardiovascular dynamics, neuromuscular activity, brain function, peripheral circulation, and movement behavior. Nevertheless, their practical implementation in dynamic sports environments remains constrained by motion artifacts, unstable sensor–skin interfaces, limited long-term wearing comfort, and insufficient model generalizability (Hu et al., 2024; Nikitina et al., 2022). Therefore, the development of sensing modalities capable of providing robust, interpretable, and multidimensional physiological information remains an important challenge in sports health monitoring.

Among the available physiological sensing technologies, human bioimpedance has attracted attention because it is non-invasive, repeatable, and compatible with portable measurement (Gersing et al., 2003). In this review, human bioimpedance is used as the general term for measuring the complex electrical response of biological tissues under externally applied alternating current. Changes in the measured impedance are associated with tissue composition, fluid distribution, electrolyte conditions, and local physiological activity (Yang et al., 2017). Human bioimpedance has therefore been investigated for body composition, hydration, fatigue, and muscle assessment (Demir et al., 2016; Lo et al., 2017). Developments in flexible electrodes, wearable devices, multi-frequency measurement, and bioimpedance spectroscopy (BIS) have improved its feasibility in exercise scenarios (Schoutteten et al., 2024; Ni et al., 2024). Nevertheless, measurement stability, physiological specificity, and cross-scenario applicability remain important limitations (Cornish et al., 1998; Hafid et al., 2024). Accordingly, this review treats human bioimpedance as a complementary measurement domain whose contribution should be evaluated using clearly defined impedance variables, analytical models, and application-specific validation (Zhou et al., 2025b).

Accordingly, this review examines human bioimpedance technologies for sports health monitoring from the perspectives of physiological basis, measurement methodology, state characterization, and emerging applications. Particular attention is given to the distinction between directly measured resistance and reactance, derived impedance magnitude and phase angle (PhA), BIA-based body-composition estimates, BIS-derived frequency-response parameters, bioelectrical impedance vector analysis (BIVA), and EIMbased local muscle assessment. Applications in body composition, hydration, fatigue, muscle function, and multimodal monitoring are reviewed with consideration of their current validation status. Body composition and hydration have been investigated more extensively, whereas fatigue and muscle-function assessment still require stronger physiological and experimental validation. The effects of motion, sweating, electrode– skin contact, posture, and muscle contraction on dynamic measurement are also discussed, together with future developments in wearable sensing, multimodal integration, and intelligent analysis.

**Figure 1:**
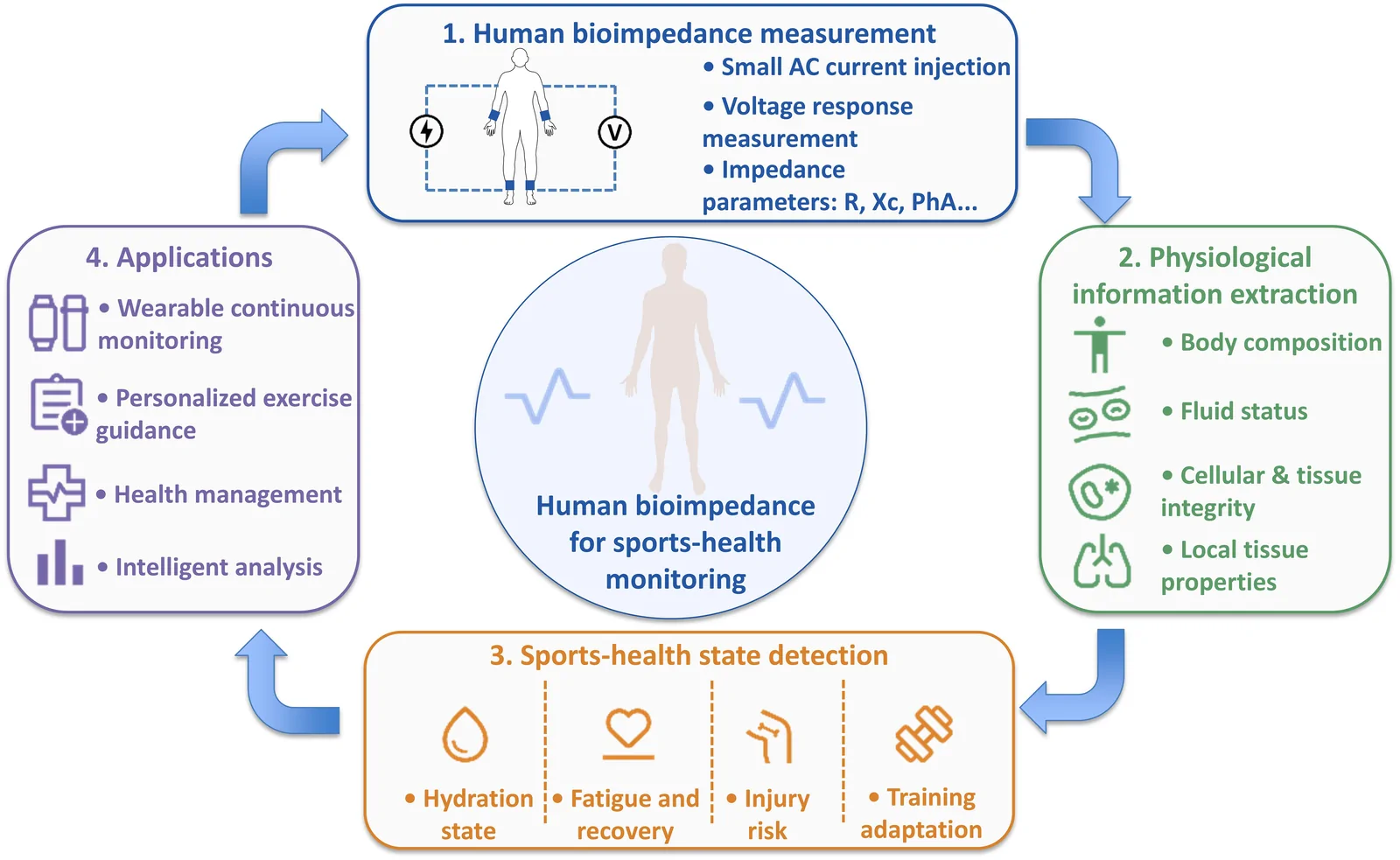
Conceptual framework of human bioimpedance for sports-health monitoring.

The conceptual framework of the review is summarized in **Figure 1**. Human bioimpedance measurement first converts the tissue response to a small alternating current into resistance, reactance, impedance magnitude, and PhA. These electrical variables are subsequently interpreted in relation to body composition, fluid status, cellular and tissue integrity, and local tissue properties. Their application-specific interpretation supports the assessment of hydration, fatigue and recovery, injury-related risk, and training adaptation, while wearable monitoring and intelligent analysis provide the basis for longitudinal and individualized sports-health management.

## 2 Literature search and review scope

A structured narrative search was conducted using Web of Science. The core search terms included “human bioimpedance”, “bioelectrical impedance analysis”, “bioimpedance spectroscopy”, “electrical impedance myography”, “phase angle”, and “sports health monitoring”, supplemented by application-specific terms related to body composition, hydration, fatigue, and muscle function. The engineering abbreviation “BioZ” was additionally retained as a search term for circuit- and wearable-system studies, but it is not treated as a separate measurement modality in this review. Most included studies were published between 2018 and 2026. Original research, validation studies, system-oriented studies, and relevant reviews were screened by title and abstract, followed by full-text assessment when necessary. Studies directly supporting human bioimpedance measurement, physiological interpretation, sports applications, or wearable implementation were retained, whereas weakly related studies were excluded.

Previous literature has established important foundations for bioimpedance measurement, athlete assessment, local muscle evaluation, and wearable sensing. However, the available reviews and representative studies generally emphasize individual methods, parameters, or application scenarios. **Table 1** summarizes these differences and the broader scope adopted in the present review.

**Table 1:** Comparison of representative previous literature and the present review.

Research focus	References	Previous scope	Main limitation	Complementary contribution
BIA/BIS fundamentals	(Kyle et al., 2004; Ward and Brantlov, 2023)	Principles, equivalent circuit models, frequency dependence, and parameter interpretation	Remain grounded in resting contexts and fail to address exercise-induced fluctuations	Extends fundamental principles to exercise-state interpretation and discusses changes in bioimpedance parameters during exercise
Athlete body composition	(Campa et al., 2021, 2022)	Validity of BIA prediction equations; athlete-specific reference values; fat-free mass estimation	Overemphasize composition estimation and neglect interactions with hydration, fatigue, and recovery	Positions body composition within a multi-state sports framework and links impedance-derived metrics to fatigue, hydration, and performance
BIVA and phase angle	(Castizo-Olier et al., 2018; Di Vincenzo et al., 2019)	BIVA vector patterns; PhA as an indicator of cellular integrity; reference ellipses	Focus on isolated parameters and insufficiently connect PhA shifts to functional sports outcomes	Provides multi-parameter synthesis by systematically mapping bioimpedance parameters to multiple sports state domains
Hydration monitoring	(Francisco et al., 2021; Aburto-Corona et al., 2024)	Fluid compartments; dehydration/rehydration assessment; hydration trends	Rely heavily on population-specific data and lack validation for individual-level monitoring during exercise	Emphasizes trend monitoring over single-point assessment and defines evidence levels for hydration monitoring during exercise
EIM and muscle assessment	(Cebrián-Ponce et al., 2021)	Local muscle properties; tissue integrity; muscle composition via localized impedance	Confine analysis to local tissues and ignore integration with systemic fatigue and recovery assessment	Bridges localized EIM findings with whole-body fatigue-recovery mechanisms and physiological state
Wearable BioZ systems	(Schoutteten et al., 2024; Ni et al., 2024)	Flexible sensing; wearable architectures; continuous acquisition; hardware design	Prioritize hardware development and underemphasize reliability validation under real exercise conditions	Provides reliability assessment under exercise conditions and addresses motion-artifact suppression with multimodal integration

Although the database, search terms, publication period, and inclusion criteria were defined to enhance the transparency of the literature retrieval process, the screening and qualitative synthesis did not follow the standardized and strictly reproducible procedures required for a systematic review. Therefore, the selection and interpretation of representative studies involved a certain degree of author judgment, which may have introduced subjective selection bias. Accordingly, the literature included in this review should be regarded as a structured qualitative synthesis of representative studies rather than an exhaustive identification of all available evidence.

## 3 State characterization and detection methods in sports health monitoring

State characterization and detection constitutes the foundation of sports health monitoring and provides the basis for understanding exercise-induced physiological responses, functional adaptations, fatigue accumulation, and recovery-related changes (Guo et al., 2019). Unlike static health assessment, human state during exercise is influenced by training load, exercise intensity, recovery level, environmental conditions, and inter-individual variability, and therefore changes continuously over time (Liaqat et al., 2022). Different state targets are associated with distinct biological mechanisms, temporal characteristics, and application purposes, which further determine the requirements for signal acquisition, sensing modalities, and analytical strategies (Zhao and Li, 2020; Zhao et al., 2022). A systematic understanding of these state targets and their corresponding detection signals is essential for improving assessment accuracy and developing personalized sports health monitoring systems.

### 3.1 Key states in sports health monitoring

In terms of specific assessment targets, sports health monitoring commonly focuses on physiological status, motor functional status, and fatigue–recovery status (Cao et al., 2024; Friedl and Looney, 2023; İlbak et al., 2026). Physiological status reflects the overall regulatory response of the body to exercise stimuli, motor functional status describes external functional performance and movement control, and fatigue–recovery status characterizes the decline and subsequent restoration of body function after exercise load (Duan and Lin, 2024). These states are closely related, but they differ in monitoring focus and information content. Distinguishing among them helps clarify the role of different sensing signals in state assessment and provides a basis for selecting appropriate monitoring technologies.

Physiological status primarily reflects systemic regulation during rest, exercise, and recovery and provides the basis for evaluating internal load and recovery-related changes (Düking et al., 2020a). Monitoring commonly focuses on cardiopulmonary regulation, thermal homeostasis, and autonomic nervous activity, which together describe how the organism responds to exercise stimuli (Takahashi et al., 2022). These indicators are most informative when interpreted longitudinally and in relation to exercise intensity, environmental conditions, and recovery stage.

**Figure 2:**
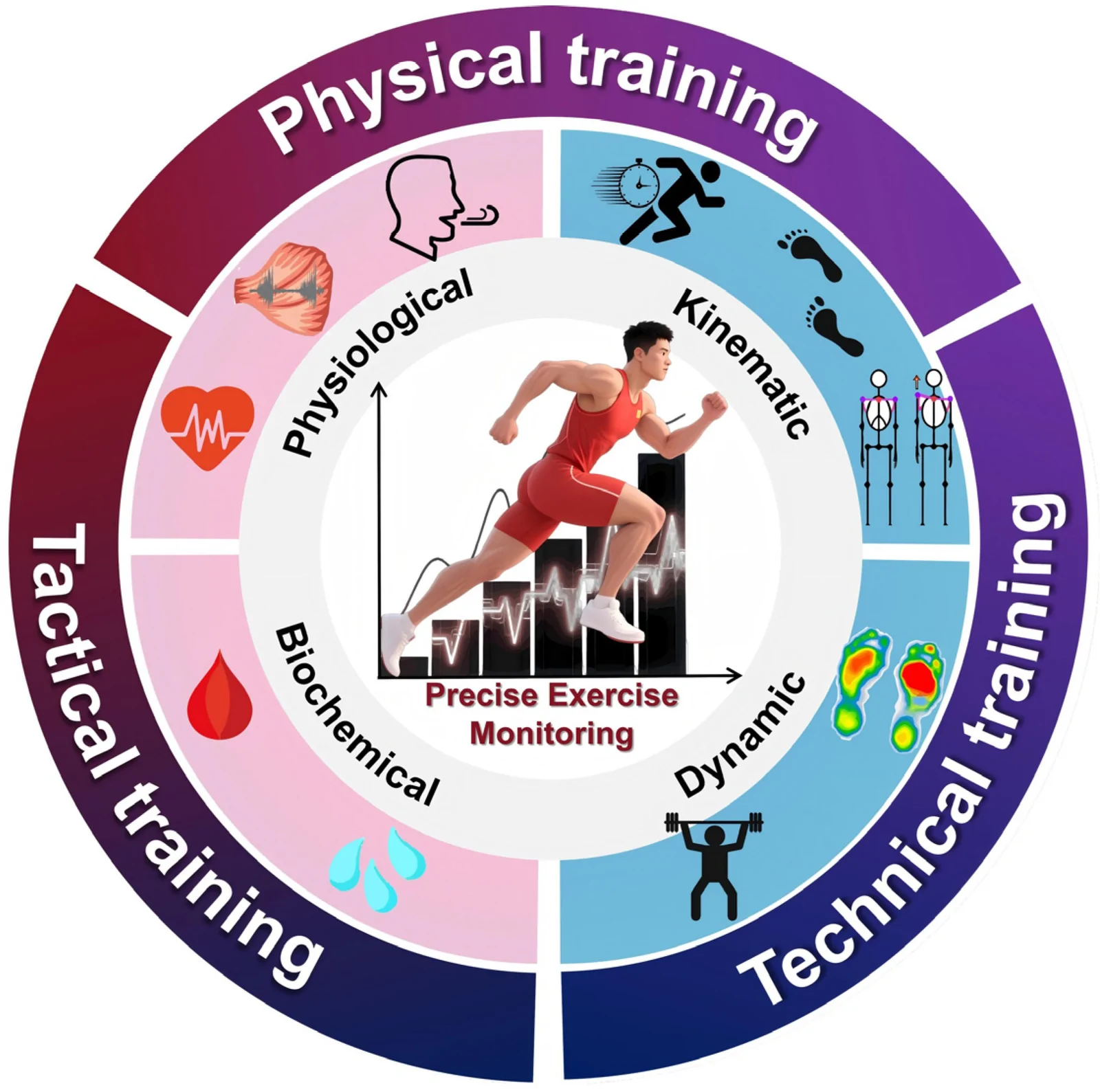
Overview of wearable sensing dimensions and representative indicators for exercise monitoring (Su et al., 2025).

Motor functional status mainly reflects functional output and movement control during motor tasks and is an important component of assessing movement performance, functional adaptation, and potential risk. Compared with physiological status, changes in motor function are more commonly manifested as alterations in movement patterns, stability, and neuromuscular coordination, which provide useful information on training adaptation and functional capacity (Shull and Jirattigalachote, 2014; Taborri et al., 2020). As summarized in **Figure 2**, wearable exercise monitoring integrates physiological, biochemical, kinematic, and biomechanical sensing dimensions. This overview positions human bioimpedance within the broader monitoring landscape and clarifies which sports-health dimensions require complementary sensing. Movement trajectories, joint motion, postural stability, and mechanical loading provide observable indicators of motor functional status, while physiological and biochemical signals contribute complementary information on exercise-related responses (Su et al., 2025). Under fatigue or exercise load, dynamic balance and movement control may also change, reflecting functional adaptation to exercise stimuli (Johnston et al., 2019). These measures therefore characterize how physiological and neuromuscular changes are expressed through movement performance and control.

Fatigue–recovery status characterizes the dynamic process of functional decline, compensatory regulation, and gradual restoration following exercise load. Fatigue should not be understood merely as reduced exercise performance, but as the result of interacting mechanisms involving neuromuscular regulation, metabolic stress, and tissue adaptation. Recovery reflects the gradual restoration of physiological homeostasis and functional capacity after exercise (Daanen et al., 2012; Manser et al., 2021). Current assessment therefore integrates indicators such as heart rate recovery, heart rate variability, EMG features, force output, changes in exercise performance, and perceived fatigue (Elshafei and Shihab, 2021). Because these indicators represent different components and time scales of the fatigue–recovery process, their combined interpretation is generally more informative than reliance on a single measure.

Overall, physiological status, motor functional status, and fatigue–recovery status represent three interconnected dimensions of sports health monitoring (Ji et al., 2025). Cardiovascular, respiratory, thermal, neuromuscular, and kinematic signals characterize different components of these dimensions (Yang and Zhao, 2025). Their assessment therefore depends on matching each sensing modality to the intended state target and combining signals when a single modality cannot resolve the required temporal or physiological dimension.

### 3.2 Conventional detection methods in sports health monitoring

**Figure 3:**
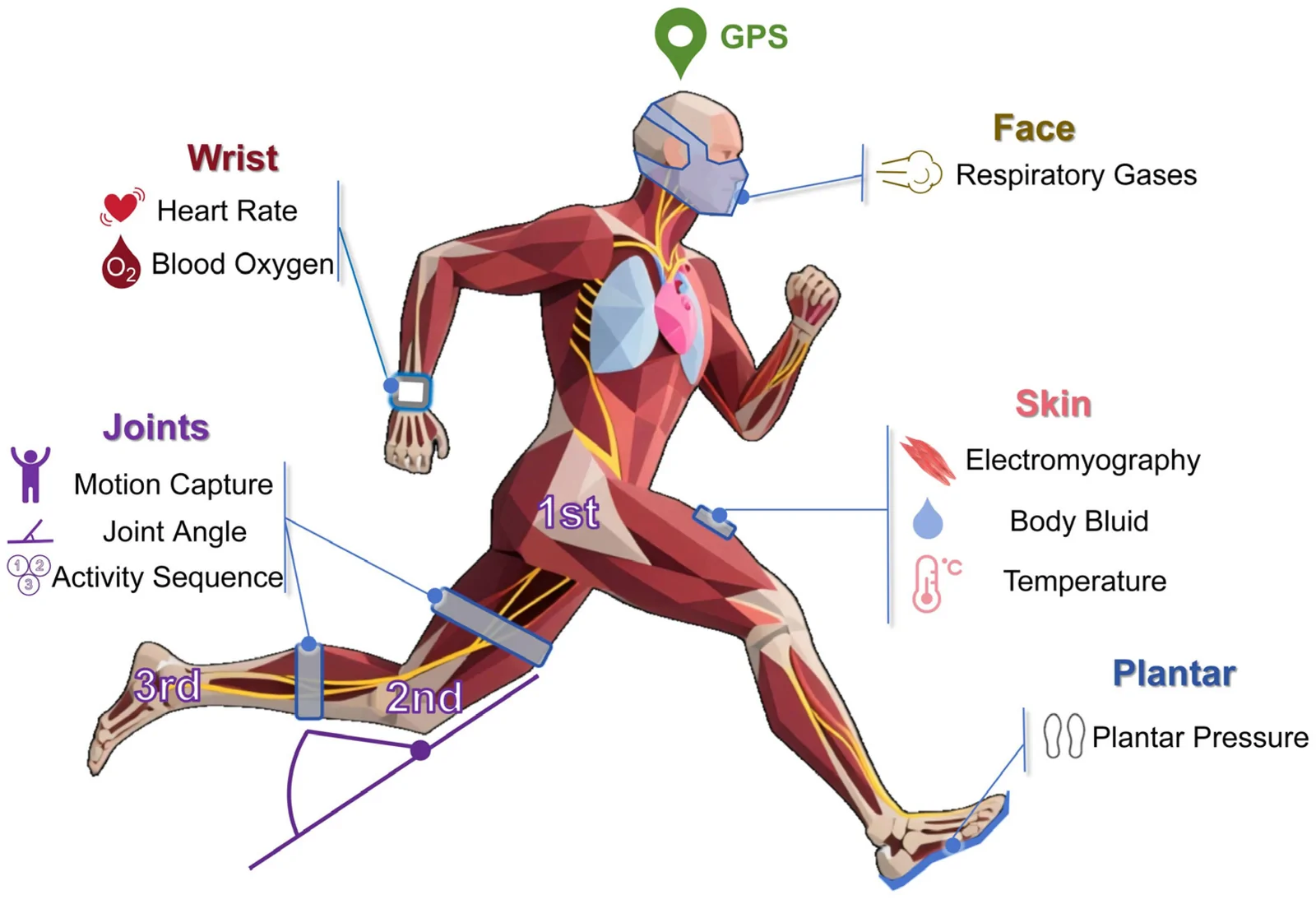
Correspondence among wearable sensing modalities, anatomical locations, and monitoring indicators (Su et al., 2025).

To meet the monitoring needs of physiological status, motor functional status, and fatigue–recovery status, sports health monitoring usually requires multisource signals to comprehensively characterize physiological and functional changes in the human body. Current wearable monitoring technologies are mainly built around different signal modalities, including electrophysiological signals (Wang et al., 2020; Zhang et al., 2023; Yin et al., 2020), optical signals (Haddad et al., 2021), and kinematic and inertial signals (Hibbing and Khan, 2024). As shown in **Figure 3**, these sensing modalities are distributed across different anatomical locations and correspond to distinct physiological or functional indicators, illustrating the relationship among sensor placement, signal source, and monitoring target in exercise assessment. This mapping is relevant because the information obtained from each modality, including human bioimpedance, is constrained by sensor location and the physiological process being targeted. Different signal modalities have distinct physiological sources and monitoring focuses, providing staterelated information from the perspectives of cardiovascular regulation, neuromuscular activity, movement execution, peripheral circulatory changes, and thermoregulatory responses. Because these signals differ in temporal resolution, resistance to interference, wearing configuration, and physiological interpretability, their applicable scenarios and informational contributions also vary.

#### 3.2.1 Physiological detection methods

Physiological detection methods are mainly used to characterize dynamic changes in the body’s internal regulatory responses during exercise loading and subsequent recovery, and they represent one of the most widely applied technical approaches in sports health monitoring. Heart rate and heart rate variability derived from ECG can reflect cardiovascular responses and autonomic nervous regulation, and are commonly used to assess exercise intensity, internal load, and recovery status (Kraik et al., 2025). PPG, as a non-invasive optical detection method, can obtain heart rate, oxygen saturation, and microcirculation-related information by detecting changes in peripheral blood volume, thereby providing an important basis for wearable continuous monitoring (Xing et al., 2023). EDA and skin temperature can further describe the body’s adaptive responses to exercise stimuli from the perspectives of sympathetic nervous activity, sweating response, and thermoregulation (Vieluf et al., 2019; Dervieux et al., 2024). When the research focus further involves neuromuscular activation, motor control, central fatigue, or exercise-related cognitive states, EMG and EEG can also be incorporated into the physiological monitoring framework to extend the characterization of peripheral muscle activity and central nervous regulation (He et al., 2026; Wu et al., 2025).

Although physiological detection methods can reflect systemic regulation and short-term physiological fluctuations during exercise, their stable application in dynamic exercise environments remains subject to certain limitations. Most physiological signals are acquired using surface-mounted sensors, and signal quality can be affected by motion artifacts, sensor displacement, interface contact conditions, sweating, and environmental factors, thereby reducing the stability and comparability of results across different exercise scenarios.

#### 3.2.2 Kinematic and biomechanical detection methods

Kinematic and biomechanical detection methods focus on evaluating motor function from the perspective of human movement behavior and its mechanical responses. By analyzing movement characteristics and loading conditions during task execution, these methods help reveal an individual’s motor control ability, coordination level, and changes in mechanical load. IMUs can continuously record acceleration, angular velocity, and posture-related information, and are commonly used to characterize gait features, limb movement trajectories, and postural stability (Losada-Benitez et al., 2023). Therefore, they have been widely applied in exercise monitoring and rehabilitation assessment (Angelucci et al., 2024). Motion capture systems can reconstruct three-dimensional human movement processes, providing detailed data support for movement technique analysis, joint kinematics research, and movement pattern recognition (Zhou et al., 2024). Meanwhile, force platforms and plantar pressure sensors can measure ground reaction forces, pressure distribution characteristics, and load transfer processes, thereby reflecting stability and mechanical output during human movement from a kinetic perspective (Zhou et al., 2025a; Li et al., 2024). When the relationship between neuromuscular activity and movement performance requires further investigation, sEMG is often combined with kinematic and kinetic data to analyze muscle recruitment strategies, movement coordination mechanisms, and fatigue-related changes (Nejadmoghbeli et al., 2024).

Compared with physiological indicator monitoring, kinematic and biomechanical detection methods mainly reflect external movement performance and its mechanical consequences, and their application effectiveness largely depends on measurement conditions and data quality. Factors such as sensor placement, calibration accuracy, task design, data processing procedures, soft tissue artifacts, individual differences in movement habits, and environmental variations may affect data stability, making consistency and comparability across studies or application scenarios difficult to ensure.

#### 3.2.3 Biochemical and metabolic detection methods

Biochemical and metabolic detection methods primarily reflect changes in the body’s internal environment during exercise loading and recovery at the molecular and metabolite levels (Jaguri et al., 2023). Their value lies in providing more mechanistic evidence for exercise-induced metabolic changes and recovery processes (Zhang et al., 2026). During exercise, the body’s energy supply pathways, glucose metabolism, and water–electrolyte status change with exercise intensity and duration. Therefore, indicators such as blood lactate, blood glucose, and electrolytes are commonly used to assess metabolic load, hydration status, and the maintenance of internal homeostasis (Zhang et al., 2021; Liu et al., 2026). In the post-exercise recovery phase, cortisol, creatine kinase, and related biomarkers derived from sweat or saliva can further reflect stress responses, the degree of muscle damage, and recovery progress (Luu et al., 2026; Satija et al., 2026). Unlike signals such as ECG, PPG, and IMU, biochemical and metabolic indicators provide information that is closer to the underlying molecular mechanisms. Therefore, they play an important role in fatigue analysis, training load assessment, and recovery status validation. With the development of wearable biochemical sensors, some biochemical detection approaches are gradually moving toward more portable and continuous monitoring applications, providing new technical support for multidimensional state monitoring in exercise scenarios (Ray et al., 2019; Seshadri et al., 2019). However, biochemical and metabolic detection methods are still constrained by factors such as sampling procedures, detection timeliness, and result interpretation. Traditional approaches largely rely on biofluid samples and specialized equipment. Although wearable biochemical sensing technologies have improved the convenience of detection, the stability of real-time continuous monitoring still requires further improvement. In addition, biochemical indicators are easily influenced by factors such as exercise load, nutritional status, and environmental conditions. As a result, a single indicator is often insufficient to comprehensively reflect sports health status, making these methods more suitable as complementary evidence within multimodal monitoring frameworks.

Taken together, physiological, kinematic, biomechanical, and biochemical methods form the principal basis of exercise-state monitoring and provide complementary views of regulation, performance, loading, and metabolism. Each modality nevertheless has specific constraints in continuity, motion robustness, sampling, or physiological specificity. The selection and combination of sensors should therefore be determined by the target state, exercise context, and required temporal and spatial resolution. Against this background, the following section examines human bioimpedance as a complementary modality and evaluates where impedance variables provide additional and sufficiently validated information.

## 4 Human bioimpedance for physiological state assessment

**Figure 4:**
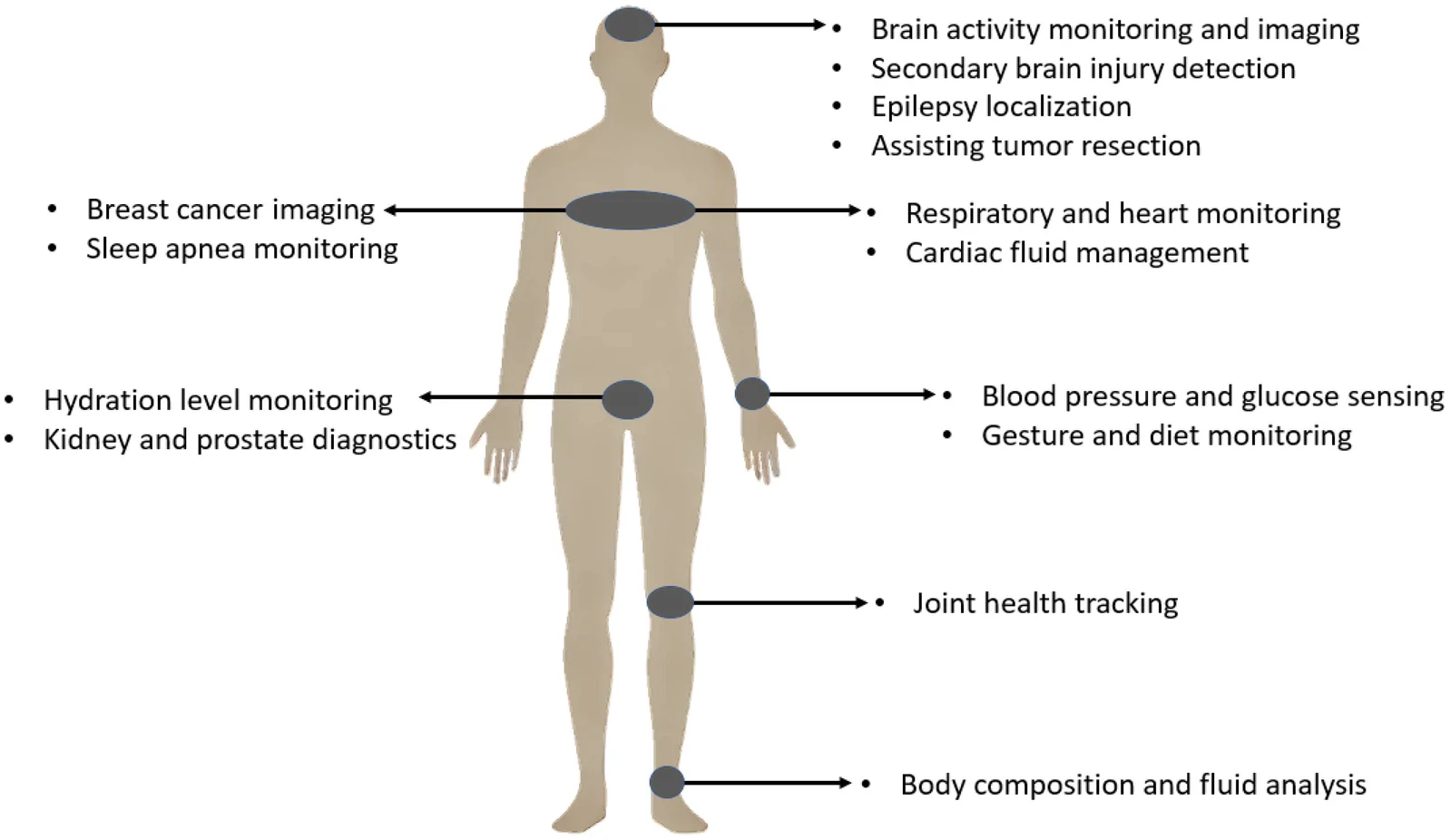
Representative applications of bioimpedance measurement (Arabsalmani et al., 2025).

In sports health monitoring, the contribution of human bioimpedance depends on how measured electrical variables are linked to specific assessment targets. At a specified frequency, complex impedance comprises resistance and capacitive reactance, while impedance magnitude and PhA are derived from these components. These related but non-interchangeable variables vary with tissue composition, fluid distribution, membrane-associated polarization, and regional geometry. As summarized in **Figure 4**, measurements obtained from different anatomical regions have been applied to body composition, hydration, cardiopulmonary, and local muscle assessment. The figure connects electrode-defined measurement regions with the application categories reviewed below, helping to distinguish whole-body, segmental, and localized interpretations. Their physiological interpretation nevertheless depends on measurement location, electrode configuration, excitation frequency, posture, and application context (Arabsalmani et al., 2025; Ren et al., 2021). This framework provides the measurement basis for the application-specific analyses that follow.

### 4.1 Complementary role of human bioimpedance in emerging wearable monitoring

Recent advances in flexible sensing interfaces, wearable platforms, and intelligent analysis have improved the concurrent acquisition of multiple physiological and movement signals during exercise (Liu et al., 2020; Rajan et al., 2018). The practical question is therefore not whether one modality offers a universally more complete description, but how signals with different physiological origins, spatial sensitivities, and temporal characteristics can be combined for a defined monitoring objective (Khan et al., 2020; Ni et al., 2020; Düking et al., 2020b).

Within emerging wearable systems, human bioimpedance should be positioned as a complementary rather than substitutive modality. IMUs and sEMG characterize movement and muscle activation, whereas impedance variables arise from current flow through the electrode-defined tissue volume (Edwards et al., 2023; Felici and Del Vecchio, 2020). The relevant question is whether resistance, reactance, impedance magnitude, or PhA contribute information beyond that explained by posture, movement, and concurrent physiological signals. Reported associations of PhA and localized impedance with muscle quality, physical activity, and fatigue-related status suggest such potential (Sardinha and Rosa, 2023; Yamada et al., 2022; Dalton-Alves et al., 2025), but these variables are not direct measures of cellular integrity or functional performance. Measurement principles, acquisition conditions, and analytical assumptions must therefore be specified before their contribution is interpreted.

### 4.2 Human bioimpedance measurement and state characterization

**Figure 5:**
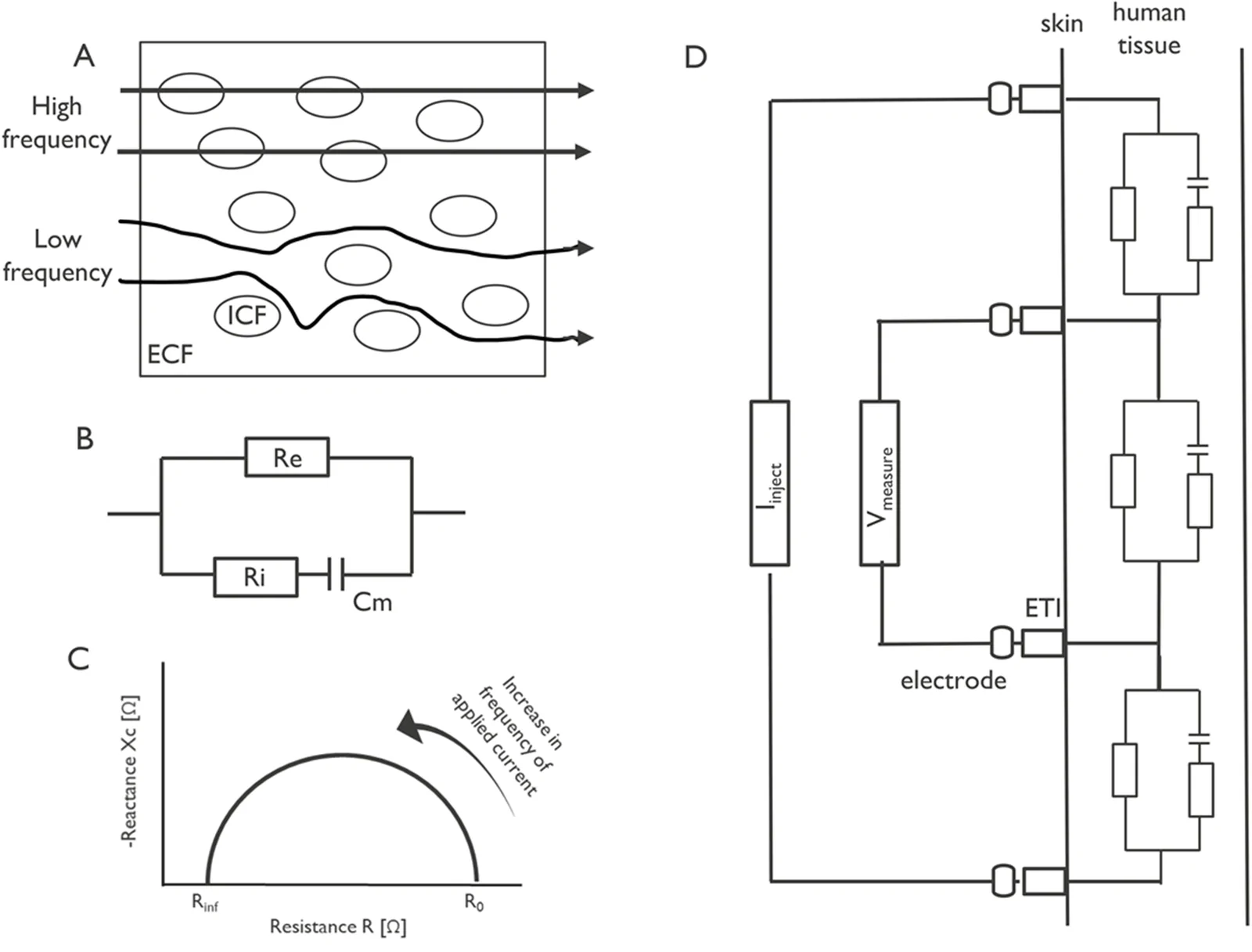
**(A)** Low-frequency current predominantly follows extracellular pathways, whereas higher-frequency current increasingly accesses intracellular conductive compartments. **(B)** Equivalent tissue model comprising extracellular resistance (*R*_e_), intracellular resistance (*R*_i_), and membrane capacitance (*C*_m_). **(C)** BIS data represented in the *R*–*X*_c_ plane, with increasing excitation frequency progressing counterclockwise. **(D)** Tetrapolar electrode configuration for bioimpedance measurement. ECF: extracellular fluid; ETI: electrode–tissue impedance; ICF: intracellular fluid; *I*_inject_: injected current; *V*_measured_: measured voltage (Groenendaal et al., 2021).

Human bioimpedance measurement is typically performed by applying a low-amplitude alternating current to the body surface while synchronously recording the resulting voltage. The voltage-to-current ratio defines the complex impedance, expressed here as *Z* = *R*−*jX*_c_, where *R* is resistance and *X*_c_ denotes the positive magnitude of capacitive reactance. Impedance magnitude |*Z*| and phase angle (PhA), calculated from the relationship between *R* and *X*_c_, are derived rather than independent measurement variables. Bioelectrical impedance analysis (BIA) combines these electrical variables with anthropometric information and prediction equations to estimate total body water, fat-free mass, and related body-composition indices (Kyle et al., 2004). The measured electrical response itself reflects frequency-dependent tissue conduction and polarization. Low-frequency current predominantly follows extracellular pathways, whereas higherfrequency current increasingly accesses intracellular conductive compartments because the capacitive opposition of cell membranes decreases with frequency (Ward and Brantlov, 2023). Equivalent-circuit models represent these behaviors using resistive components for conductive pathways and capacitive or reactive components for membrane-associated and interfacial polarization. **Figure 5** brings these current pathways, circuit elements, spectral representation, and tetrapolar configuration together, providing a common physical basis for the measurement approaches discussed below.

Human bioimpedance measurements are primarily influenced by excitation frequency and measurement region. Single-frequency BIA applies an analytical model to impedance obtained at one frequency, whereas multi-frequency BIA uses a limited set of discrete frequencies to improve body-fluid and composition estimates. Bioimpedance spectroscopy (BIS), by contrast, acquires impedance over a broader frequency range and commonly applies equivalent-circuit or Cole-model fitting to derive frequency-dependent parameters (Montgomery et al., 2025). Multi-frequency BIA and BIS are therefore related but should not be used interchangeably. Whole-body configurations are commonly used for body composition and total body water, segmental configurations assess regional differences, and localized measurements target a specific muscle or tissue region (Castizo-Olier et al., 2018). Interpretation is further affected by measurement protocols, electrode–skin interface conditions, immediate physiological state, and model assumptions. Postural changes can shift impedance readings (Lyons-Reid et al., 2021), and electrode type can introduce variability, particularly for PhA (Dupertuis et al., 2022). Analytical models should therefore be reported separately from the raw variables *R*, *X*_c_, |*Z*|, and PhA.

The state-characterization capability of human bioimpedance encompasses fluid status, tissue composition, and local muscle condition. Under standardized frequency and geometry, measured resistance is influenced by the effective volume and ionic conductivity of the tissue current path; it should not be interpreted as a direct or compartment-specific measure of extracellular water. Model-derived extracellular resistance may additionally be sensitive to tissue sodium content and extracellular conductive conditions (Mitsides et al., 2020). PhA has been associated with the balance between resistive conduction and membrane-related polarization, intracellular-to-extracellular fluid distribution, and overall physical status, but it is not a direct measurement of cellular integrity (Di Vincenzo et al., 2019). Studies in young elite soccer players indicate that impedance patterns and PhA are related to body composition and maturation status (Campa et al., 2019). Electrical impedance myography (EIM) specifically uses localized impedance measurements to characterize skeletal muscle and neuromuscular tissue (Mul et al., 2018); it should be distinguished from segmental BIA, which remains oriented toward regional body-composition estimation. Local impedance signals also vary with muscle contraction level, indicating potential for functional-state assessment (Coutinho et al., 2020). Flexible electrodes, miniaturized analog front-ends, and motion-robust processing have improved the feasibility of ambulatory human bioimpedance measurements (Piuzzi et al., 2020), although parameter selection and interpretation must remain specific to the measurement framework and sports task.

## 5 Applications of human bioimpedance in sports health monitoring

As human bioimpedance has gradually developed from static measurement toward exercise-oriented and continuous assessment, its role in sports health monitoring has extended beyond conventional body composition analysis. Fluid regulation, fatigue-related changes, muscle condition, and multisource physiological monitoring have increasingly become important application targets, while the interpretation of impedance parameters remains influenced by sport-specific characteristics and measurement conditions (Di Vincenzo et al., 2019; Abdelnour et al., 2024). Current research shows different levels of methodological development and validation across these application scenarios. This section reviews the applications of human bioimpedance in body composition assessment, hydration and fatigue monitoring, muscle function evaluation, and multimodal monitoring (Umer et al., 2025; Yu et al., 2025).

To provide a structured comparison of the evidence supporting major sports-health applications, **Table 2** summarizes representative original studies on body composition, hydration, fatigue-related responses, and muscle function. The comparison reports participant characteristics, measurement frequency, electrode placement, impedance variables, comparator methods, principal findings, and study-specific limitations. The studies were selected to represent both established whole-body applications and emerging localized assessments rather than to provide an exhaustive catalogue of all available investigations.

**Table 2:** Representative studies using human bioimpedance for body composition, hydration, fatigue-related responses, and muscle function in physically active populations.

Application		Study		Population and sample size		Measurement method and frequency		Electrode placement		Key impedance parameters		Comparator or reference method		Major findings		Main limitations	
Body composition and hydration		Martins et al., 2021		University athletes from team and individual sports; *n* = 167		Multi-frequency BIA; impedance at 1, 50, 250, 500, and 1000 kHz; BIVA based on 50 kHz data		Eight contact electrodes at both hands and feet		*R*. *X*_c_, PhA, *R*/*H*, and *X*_c_/*H*		Non-athlete BIVA reference population and sport-group comparison		Most athletes were located within the 50% tolerance ellipse; vectors were shifted leftward relative to non-athletes, consistent with higher body cell mass		Cross-sectional design; convenience sample; interpretation depended on an external reference population	
Body composition		Campa et al., 2020		Male professional volleyball, soccer, and rugby players; *n* = 164		Phase-sensitive single-frequency BIA; 50 kHz		Whole-body tetrapolar hand- foot configuration in the supine position		*R*. *X*_c_, PhA, vector length, *R*/*H*, and *X*c/*H*		Anthropometry, somatotype, urine specific gravity, and athlete-specific total-body-water estimates		Higher body-water content shortened the impedance vector; PhA was positively associated with mesomorphy and inversely associated with ectomorphy		Male-only sample; cross- sectional analysis; findings limited to single-frequency devices and resting conditions	
Hydration and fluid distribution		Francisco et al., 2020		Athletes from endurance, power, and team sports; *n* = 273 (202 men and 71 women)		Whole-body BIS; raw variables evaluated at 50 kHz		Four electrodes on the right hand/wrist and right foot/ankle; supine		*R*. *X*_c_, PhA, *R*/*H*, and *X*_c_/*H*		Deuterium dilution for total body water, bromide dilution for extracellular water, and DXA for body composition		PhA predicted intracellular water and extracellular-to-intracellular water distribution; resistance was strongly related to total and extracellular water pools		Cross-sectional associations do not establish individual change sensitivity; measurements were obtained under standardized resting conditions	
Longitudinal hydration and training adaptation		Francisco et al., 2024		Athletes assessed during preparatory and competitive periods; *n* = 108		Whole-body BIS at 50 kHz; series variables measured and mathematically transformed to a parallel model		Tetrapolar whole-body configuration with current and voltage electrodes separated by at least 5 cm		Series and parallel *R*/*H*, *X*_c_/*H*, *Z*/*H*, and capacitance		Deuterium dilution for total body water, bromide dilution for extracellular water, and calculated intracellular water		Changes in parallel reactance and capacitance predicted changes in intracellular water; resistance and impedance tracked total and extracellular water across the season		Two measurement periods; model transformation adds analytical assumptions; broader sport-specific validation remains necessary	
Fatigue- related local muscle response		Li et al., 2016		Healthy adults; *n* = 19		Localized EIM during graded isometric elbow flexion and sustained contraction; 1 kHz-10 MHz, with analysis at 50 and 100 kHz		Handheld linear electrode array over the dominant biceps brachii, aligned with muscle fibres		Local *R*, *X*_c_, and PhA		Elbow-flexion torque, percentage maximum voluntary contraction, and time to task failure		Resistance increased with higher contraction intensity and decreased during sustained fatigue, whereas reactance showed limited change		Small healthy cohort; single muscle and isometric task; local geometry and metabolite accumulation could not be separated	
Muscle strength and power		Fukuoka et al., 2022		Resistance-trained men; *n* = 44		Whole-body BIS: 50 kHz raw variables		Four electrodes on the dorsal right hand/wrist and right foot/ankle; supine		*R*. *X*_c_, PhA, and BIS-derived water compartments		DXA, bench-press and back-squat 1RM, Wingate test, and countermovement jump		Higher PhA was associated with countermovement-jump power and bench-press strength after adjustment, although lean soft tissue remained the strongest predictor		Cross-sectional design; modest sample; men only; associations do not define diagnostic thresholds	
Muscle power and strength		Oliveira et al., 2023		Physically active young women; *n* = 30		Whole-body and localized BIA; 50 kHz		Whole-body hand- foot pathway and localized lower-limb muscle configuration		Whole-body PhA and muscle-localized PhA		Wingate test, isokinetic dynamometry, and DXA		Whole-body and localized PhA showed associations with muscle-power outcomes, but neither measure was consistently associated with strength		Small female-only cohort; cross-sectional design; localized PhA was influenced by fat mass and measurement region	
Localized muscle measurement reliability		Longo et al., 2025		Healthy adults; *n* = 50 (25 women and 25 men)		Multi-frequency EIM at 5, 50, 100, and 200 kHz; intra- and inter-day testing		Tetrapolar adhesive electrodes over whole, proximal, and distal anterior-thigh regions		Impedance magnitude across frequencies; *R*, *X*_c_, and PhA at 50 kHz		Repeated-measure reliability and regional/sex comparisons		Measurements showed excellent reliability, while substantial regional and sex differences demonstrated the importance of electrode placement and reference stratification		Healthy resting sample; no exercise-performance or imaging comparator; subcutaneous tissue effects were not directly quantified	

BIA, bioelectrical impedance analysis; BIS, bioimpedance spectroscopy; BIVA, bioelectrical impedance vector analysis; DXA, dual-energy X-ray absorptiometry; EIM, electrical impedance myography; H, height; PhA, phase angle; R, resistance; X_c_, reactance; 1RM, one-repetition maximum.

The summarized evidence illustrates that whole-body body-composition and hydration applications have comparatively stronger validation because raw impedance variables have been examined against dilution techniques, DXA, anthropometry, and established reference distributions. By contrast, fatigue-related and muscle-function applications are supported mainly by cross-sectional associations, controlled contraction protocols, or short-term reliability studies. Their interpretation remains more dependent on muscle region, electrode geometry, contraction state, exercise protocol, and measurement timing. These differences support the use of human bioimpedance as a repeated and context-specific monitoring tool while indicating that universal diagnostic thresholds for fatigue or muscle function are not yet justified.

### 5.1 Body composition assessment

Body composition assessment is one of the most established applications of human bioimpedance in sports health monitoring. Lean tissue, adipose tissue, and body-fluid compartments contribute differently to athletic performance, training adaptation, and health status, whereas body mass and body mass index cannot identify the physiological source of an observed change (Duren et al., 2008). Human bioimpedance measurement yields resistance and reactance, from which impedance magnitude and PhA are derived. BIA combines these variables with anthropometric information and prediction equations to estimate fat-free mass, fat mass, and body water. BIVA, in contrast, evaluates height-normalized resistance and reactance as a vector without converting them directly into compartment estimates, while PhA summarizes their angular relationship. The rapid acquisition, non-invasive implementation, and suitability for repeated measurements make these approaches valuable for field-based and longitudinal assessment in sports populations (Moon, 2013). Their interpretation, however, depends on whether the physiological assumptions and reference data underlying each analytical method are appropriate for the population being examined.

**Figure 6:**
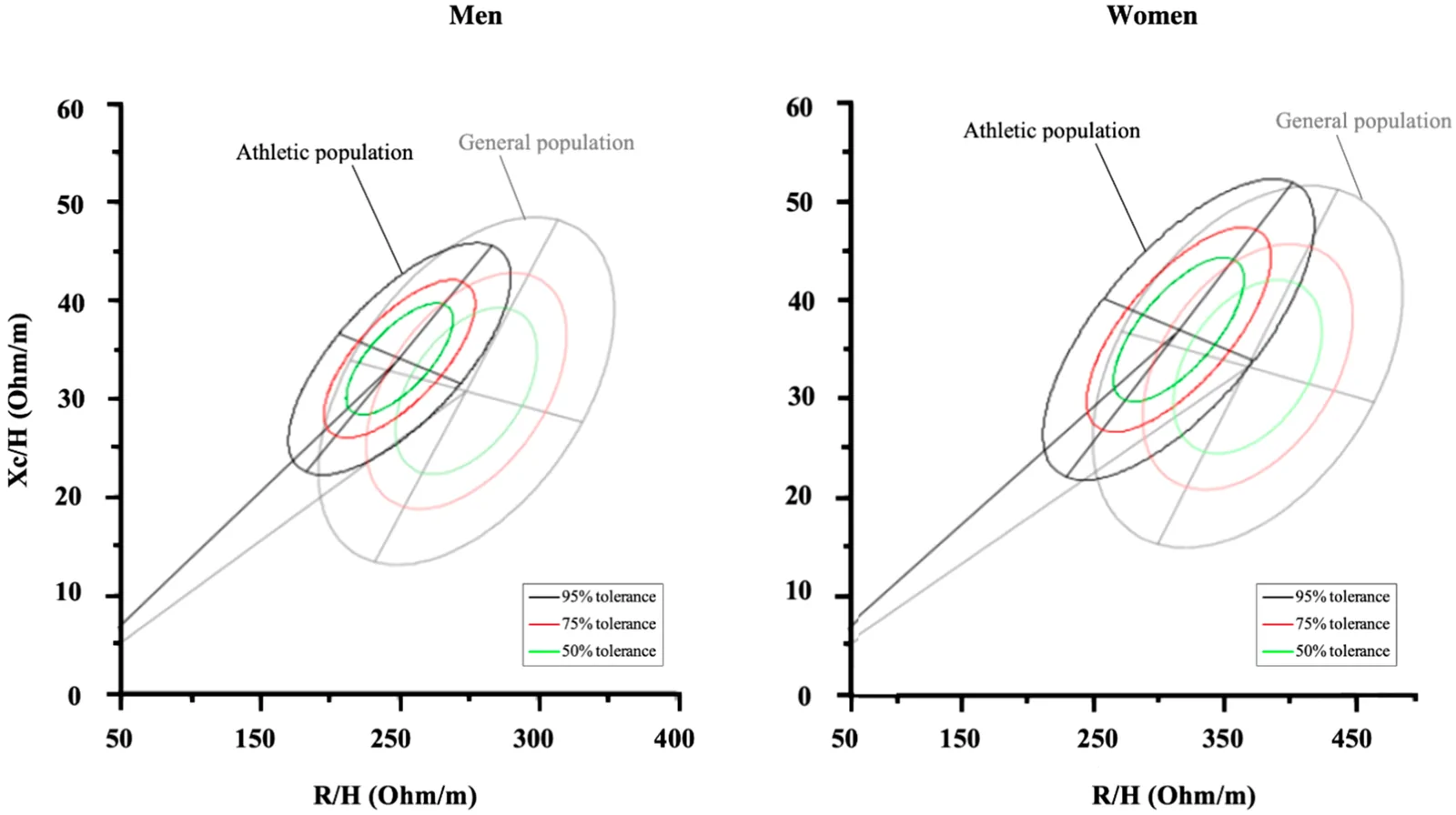
The reference tolerance ellipses for general and athletic populations are shown (Campa et al., 2021).

When impedance measurements are translated into body composition estimates, the validity of the analytical model becomes dependent on the population used for calibration. Long-term training alters regional muscularity, tissue distribution, and the hydration characteristics of fat-free tissue, thereby modifying the relationship between measured impedance and the body compartment being estimated. Models developed in general populations may consequently introduce systematic deviations when applied to trained individuals. The displacement between the general-population and athletic reference ellipses shown in **Figure 6** illustrates this distinction. Compared with the general reference distribution, the athletic ellipse is shifted toward lower height-normalized resistance and relatively higher reactance, reflecting greater conductive tissue volume and different membrane- and fluid-related properties in trained populations (Campa et al., 2021). This visual comparison supports the use of athlete-oriented analytical models and reference distributions, while also showing why the athletic ellipse should not be interpreted as a single impedance profile shared by all athletes. Measurement standardization improves repeatability, whereas population-matched calibration and reference values determine whether the resulting estimates can be interpreted appropriately (Campa et al., 2022).

Differences in BIA-derived estimates and bioimpedance profiles among sporting populations reflect the combined effects of sport-specific tissue adaptation and fluid regulation. Endurance training is commonly associated with high training volume and recurrent changes in fluid balance, which mainly influence resistance and impedance-vector length through variations in conductive fluid volume. These changes may affect estimates of total body water and fat-free mass. In contrast, strength- and power-oriented training generally promotes greater regional muscularity, increased glycogen-associated water storage, and altered intracellular fluid distribution, leading to distinct reactance, PhA, and impedance-vector characteristics (Abdelnour et al., 2024). The relationship between impedance and body composition is also influenced by training status and development. Pronounced muscular hypertrophy in bodybuilders leads to different impedance distributions from those of conventional resistance-trained individuals, while maturation-related changes in body geometry and tissue composition modify responses in younger athletes (Petri et al., 2023; Campa et al., 2019). BIA-based assessment should therefore interpret model-derived estimates together with the underlying impedance variables and the physiological characteristics of the target population.

### 5.2 Hydration and fatigue monitoring

Human bioimpedance provides a practical approach for monitoring hydration status and fatigue because exercise-induced physiological stress continuously modifies tissue electrical properties. During exercise, sweating, cardiovascular regulation, and metabolic activity jointly influence body-fluid distribution and tissue homeostasis. These processes alter intracellular and extracellular fluid balance, modify ionic conductive pathways, and affect membrane-associated polarization, resulting in changes in resistance, reactance, and PhA. These variables and impedance-vector characteristics provide information related to body composition, fluid regulation, and exercise-induced tissue adaptation (Matias et al., 2019; Francisco et al., 2021). Their responses during exercise therefore represent the integrated effects of fluid regulation and tissue changes under dynamic physiological conditions rather than a single physiological process.

**Figure 7:**
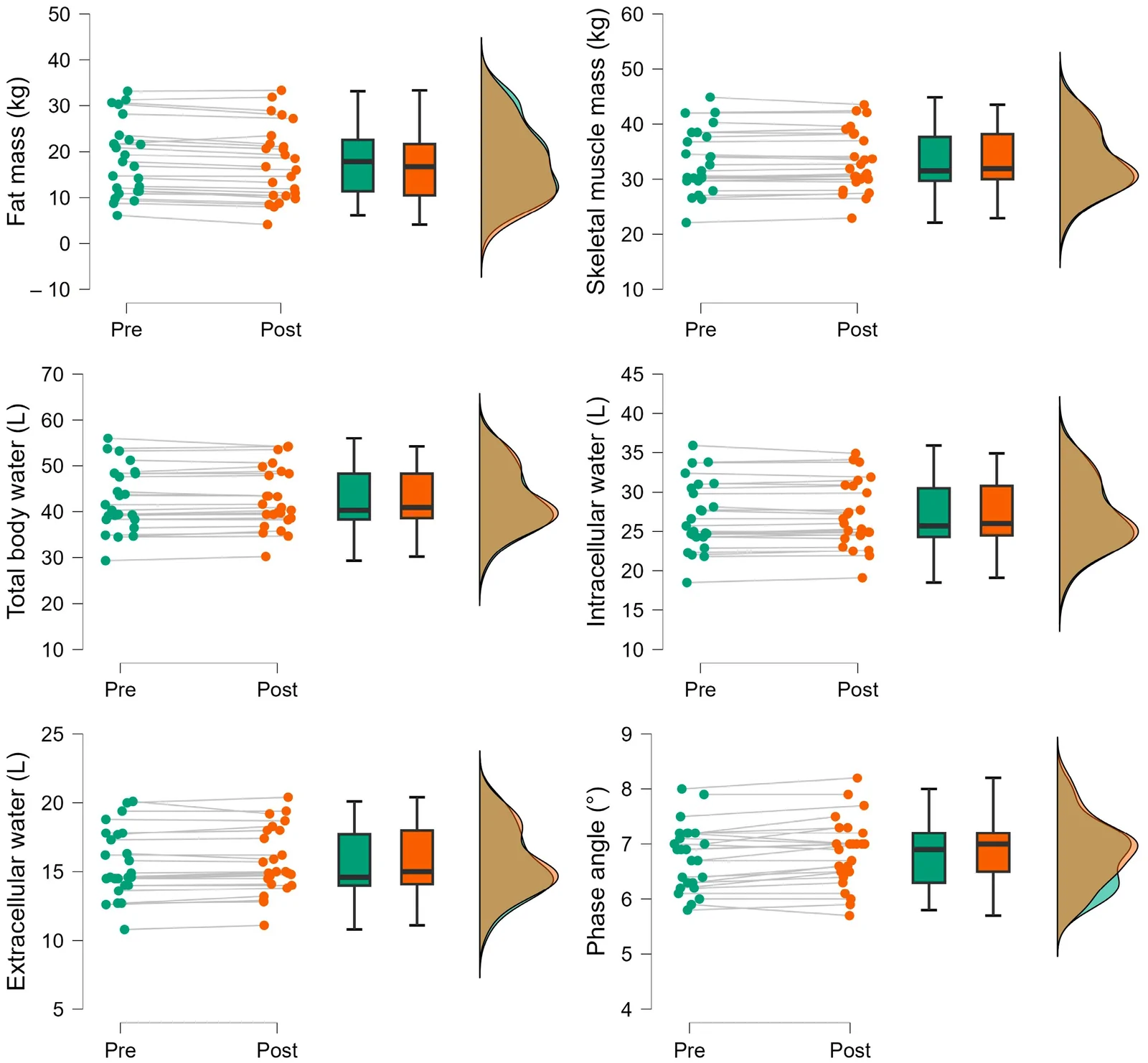
Raincloud plots showing individual and group changes in fat mass, skeletal muscle mass, total body water, intracellular water, extracellular water, and PhA before and after five hours of passive dehydration under thermal stress in males (*n* = 25) (Aburto-Corona et al., 2024).

Changes in hydration status constitute one of the primary determinants of exercise-related impedance variation. Under fixed frequency, posture, and electrode geometry, a reduction in conductive fluid volume commonly increases resistance by narrowing ionic current pathways. Resistance is therefore sensitive to effective conductive volume and ionic conductivity, but it does not directly quantify extracellular water by itself. BIA and vector-based approaches have been used to examine the relationship between body-water distribution and impedance, with vector displacement providing combined information on hydration-related changes and body-cell characteristics (Marini et al., 2020). Variations in intracellular hydration can also alter cell volume and membrane-associated polarization, contributing to changes in reactance and PhA. As illustrated in **Figure 7**, passive dehydration is accompanied by systematic changes in impedance-derived variables, indicating that human bioimpedance captures the combined effects of fluid loss and redistribution. The simultaneous presentation of individual and group responses is valuable because it highlights interindividual variability and supports longitudinal interpretation rather than reliance on a single threshold. Longitudinal vector analysis has further characterized responses during exercise and recovery, supporting individual trend monitoring under standardized conditions (Aburto-Corona et al., 2024).

Beyond acute fluid regulation, prolonged or high-intensity exercise induces metabolic stress that contributes to fatigue-related impedance changes (Fu and Freeborn, 2018; Ozmen et al., 2023). During repeated muscle contractions, glycogen depletion, ion flux, and metabolic by-product accumulation alter osmotic gradients and drive intracellular–extracellular fluid exchange (Taniguchi et al., 2020). Acute cell swelling can expand intracellular conductive volume and reduce resistance, while membrane deformation and altered ionic gradients may modify reactance by changing membrane-associated and interfacial polarization. Because PhA is determined by the relative relationship between reactance and resistance, its direction depends on the proportional change in both components. A reduction in resistance with preserved or increased reactance raises PhA, whereas a proportionally greater reduction in reactance lowers it. In later stages, inflammatory fluid accumulation and structural damage may increase regional conductivity and reduce measured resistance in the exercised region (Shiose et al., 2019). Fatigue-related impedance responses thus represent a convergent outcome of metabolic stress, fluid redistribution, tissue geometry, and membrane-related electrical changes. They should be interpreted alongside exercise load, performance changes, and recovery markers rather than as standalone indicators.

### 5.3 Muscle function assessment

**Figure 8:**
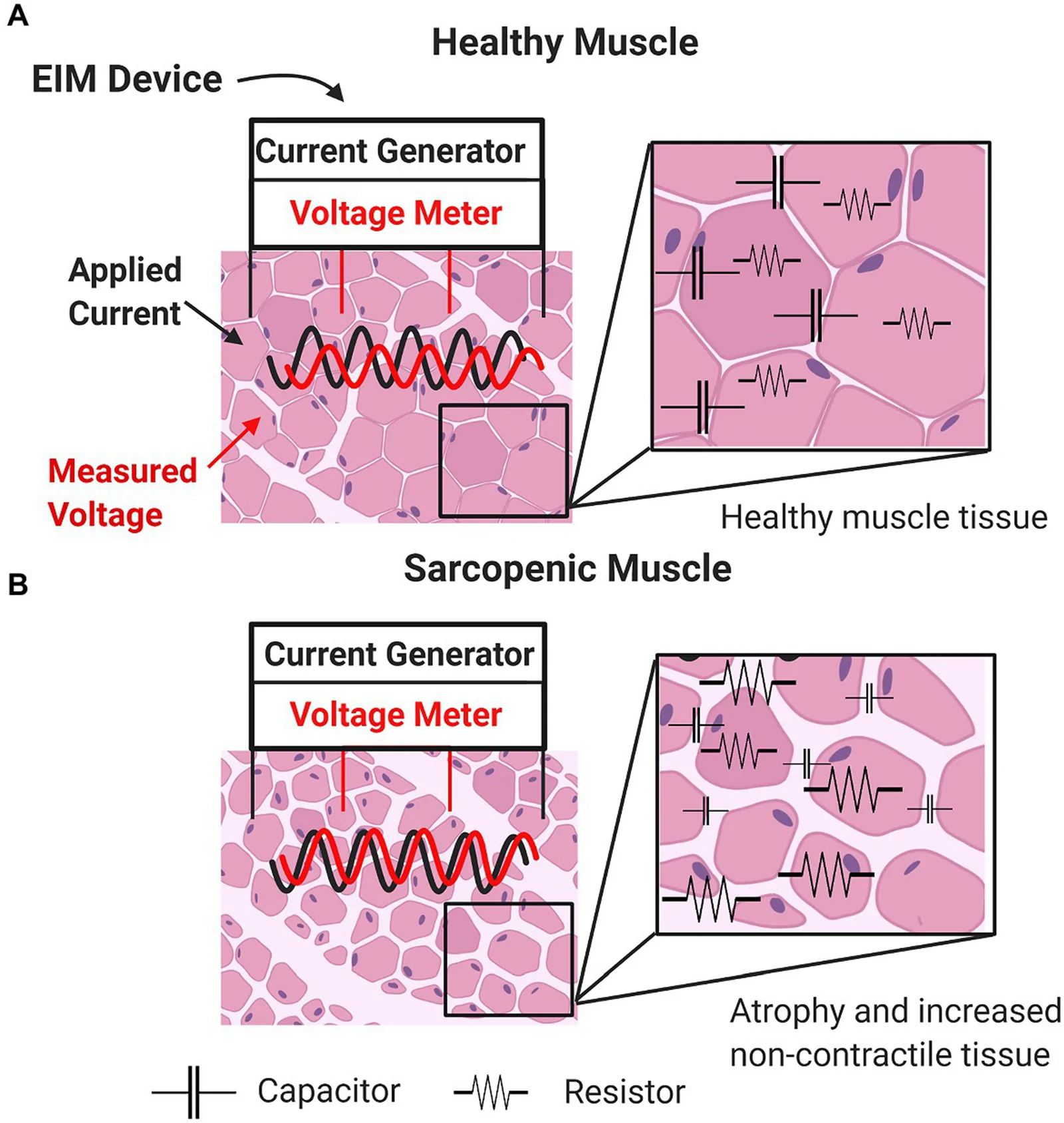
Basic concepts underlying impedance measurements of healthy muscle **(A)** and sarcopenic muscle **(B)** characterized by increased non-contractile tissue (e.g., increased myosteatosis) and smaller myocytes (i.e., atrophied myofibers) (Clark et al., 2021).

In muscle-function assessment, human bioimpedance complements the estimation of lean-tissue quantity by characterizing regional muscle structure and electrical behavior. EIM uses localized electrode configurations to assess the frequency-dependent impedance of skeletal muscle and neuromuscular tissue, whereas segmental or regional BIA applies body-composition-oriented models to a defined body segment. Although both approaches may report resistance, reactance, and PhA, they differ in current path, measurement objective, electrode arrangement, and interpretation model. EIM has shown sensitivity to muscle atrophy, fat infiltration, and detraining-related tissue alterations, as illustrated in **Figure 8** (Cebrián-Ponce et al., 2021; Clark et al., 2021). The side-by-side schematic makes the relationship between altered muscle composition and electrical current pathways explicit, supporting mechanistic interpretation rather than simple group discrimination. A reduction in water-rich contractile tissue changes the conductive crosssectional area and current pathways, whereas alterations in fiber organization and membrane interfaces influence capacitive behavior. Human bioimpedance therefore provides an indirect assessment of tissue conditions associated with force generation rather than a direct measurement of contractile performance.

**Figure 9:**
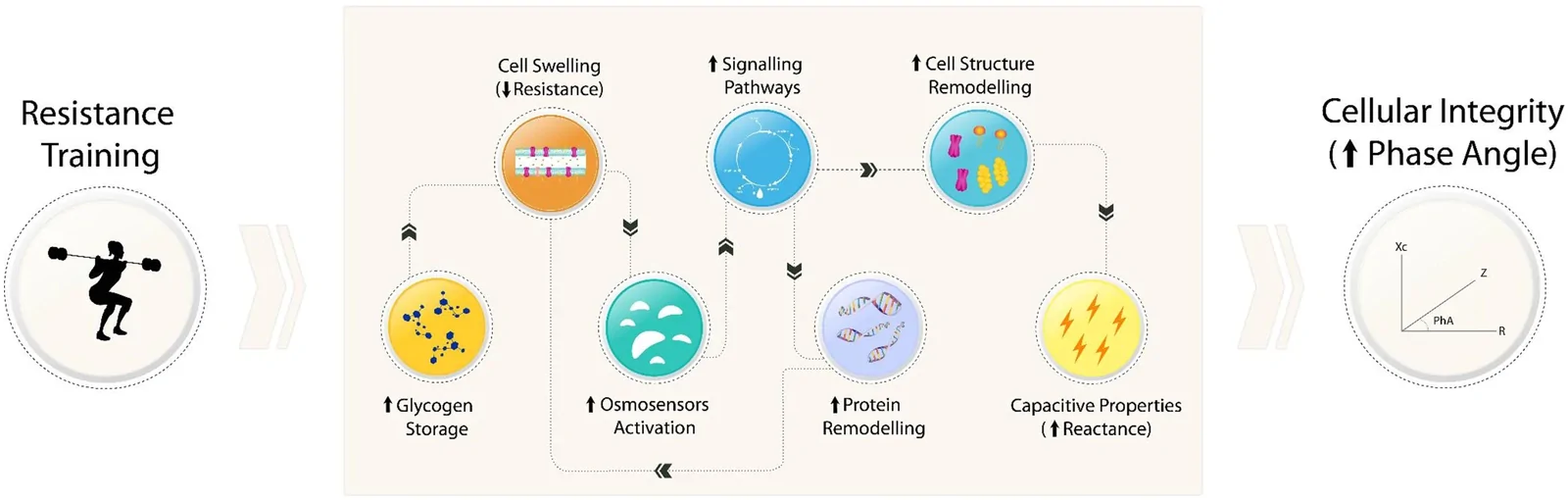
Physiological scheme illustrating the proposed effect of resistance training on bioimpedance-derived PhA (Sardinha and Rosa, 2023).

Chronic resistance-training adaptation provides a physiological basis for the associations between PhA and strength-related variables. Muscle hypertrophy increases the amount and cross-sectional area of conductive lean tissue, while greater glycogen storage is generally accompanied by increased intracellular water. These adaptations can reduce resistance by expanding ionic current pathways. Maintenance or enlargement of the electrically active membrane interface may also preserve or increase reactance. Under the same frequency, posture, and electrode geometry, an increase in reactance relative to resistance produces a higher PhA, consistent with reported associations of whole-body or segmental PhA with fat-free mass, relative strength, and power (Hetherington-Rauth et al., 2021; Silvino et al., 2024). These relationships indicate that PhA reflects tissue characteristics relevant to functional performance, although it cannot be converted directly into force or power. **Figure 9** summarizes the pathway linking glycogen-associated cellular hydration, cell swelling, osmosensitive signaling, protein remodeling, and changes in impedancederived PhA (Sardinha and Rosa, 2023). Its value is to separate the proposed biological pathway from the observed association between PhA and strength-related outcomes, emphasizing that the mechanism remains inferential.

Acute loading and exercise-induced muscle damage produce electrical responses that differ from longterm adaptation. Short-term contraction can transiently alter muscle geometry, fiber orientation, local blood volume, and fluid distribution, leading to reversible changes in regional current pathways. By contrast, damaging eccentric loading can disrupt the sarcolemma and contractile structure, initiate inflammatory responses, and promote extracellular fluid accumulation. The resulting increase in regional conductivity may reduce measured resistance (Shiose et al., 2019), whereas impaired membrane-associated polarization can contribute to reductions in reactance. Multi-frequency impedance variables have also been associated with biochemical and functional indicators of exercise-induced muscle damage (Yamaguchi et al., 2024). Because resistance and reactance may exhibit different magnitudes and temporal patterns of change, the direction of PhA depends on their relative responses during tissue damage and recovery.

Although these findings establish a physiological basis for muscle-related impedance assessment, the evidence for direct functional evaluation remains limited. Associations between impedance parameters and strength or power are derived mainly from cross-sectional cohorts, short-term exercise protocols, and relatively small participant groups (Cebrián-Ponce et al., 2021; Clark et al., 2021). Regional measurements are also sensitive to electrode placement, muscle contraction state, loading protocol, and post-exercise measurement timing. Human bioimpedance is currently better suited to longitudinal within-subject monitoring and should be interpreted together with strength testing, imaging, electromyography, or biochemical indicators. Validated thresholds for muscle quality, injury severity, and performance prediction have not yet been established.

### 5.4 Multimodal monitoring

Within multimodal monitoring, human bioimpedance is most informative when impedance changes are interpreted against concurrent cardiac, respiratory, neuromuscular, and movement events rather than treated as isolated tissue markers. In cardiopulmonary applications, combining transthoracic impedance with ECG can relate respiratory or pulmonary-fluid trends to cardiac electrical timing. In exercise applications, pairing localized bioimpedance with sEMG or inertial sensing allows changes associated with muscle deformation and regional fluid redistribution to be evaluated relative to activation and movement. Multimodal integration may therefore improve source attribution and reduce ambiguity in impedance interpretation, while the independent contribution of each channel must still be demonstrated for the intended task.

**Figure 10:**
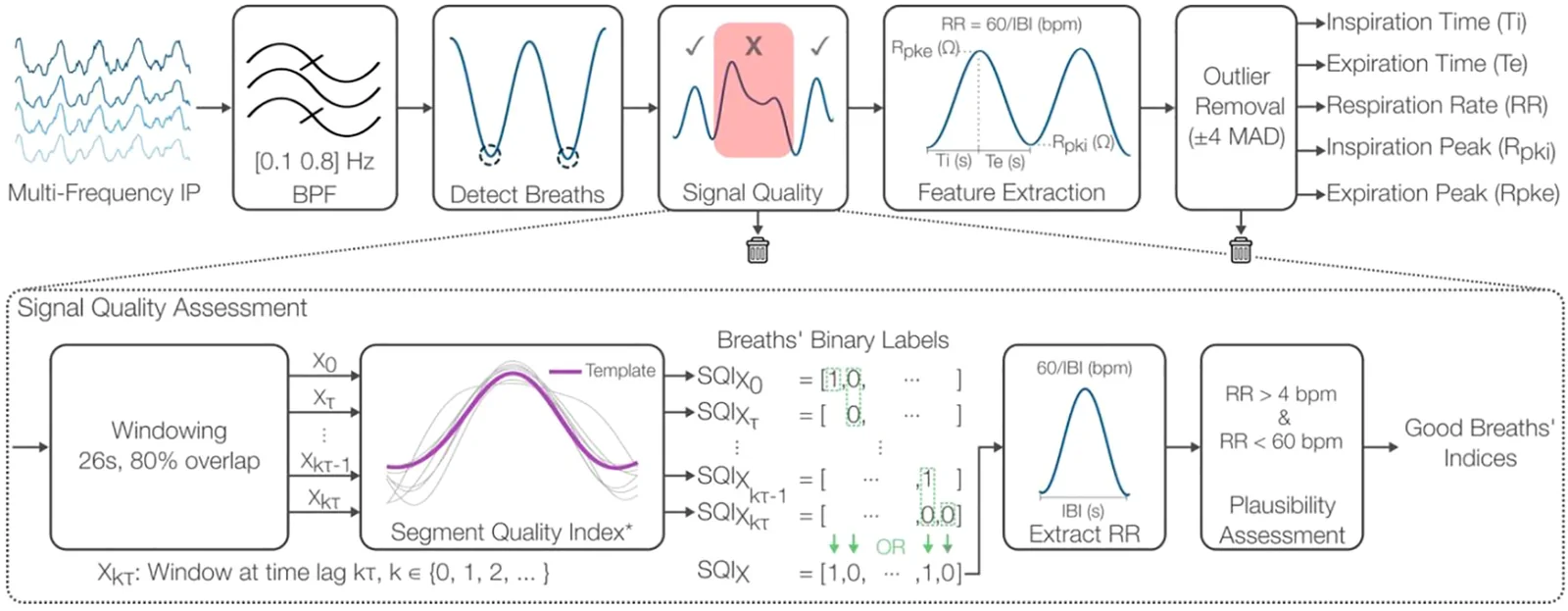
Multi-frequency impedance pneumography (IP) signal processing pipeline (Sanchez-Perez et al., 2022).

**Figure 11:**
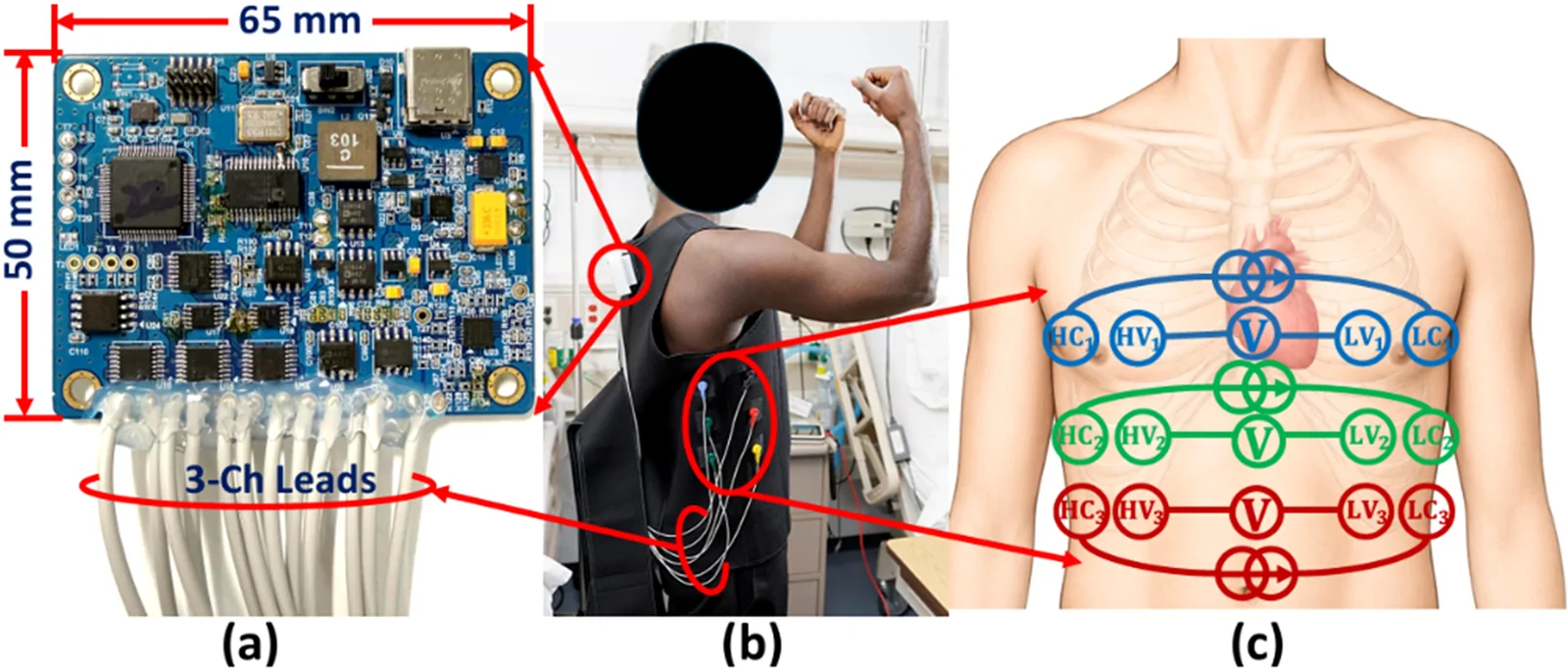
Wearable three-channel transthoracic BIS–ECG acquisition platform. **(a)** Prototype PCB; **(b)** thoracic vest with the packaged electronics module; and **(c)** three tetrapolar transthoracic electrode paths, with Path 1 additionally supporting synchronized one-lead ECG acquisition (Amin et al., 2026).

This complementary relationship has been preliminarily demonstrated in several wearable systems. A wearable platform integrating BIS, multichannel lung-sound sensing, multi-frequency impedance pneumography, temperature measurement, and kinematic sensing was developed and applied to track pulmonary fluid and respiratory changes in healthy participants and patients with heart failure (Sanchez-Perez et al., 2022). Their study showed that impedance measurements can not only provide pulmonary-fluid and respiration-related features independently, but also support cardiopulmonary assessment together with acoustic, thermal, and movement information. **Figure 10** further illustrates the processing chain from multi-frequency impedance acquisition to respiratory-feature extraction, clarifying where signal processing influences the physiological output used for monitoring. From an acquisition-architecture perspective, a wearable three-channel transthoracic BIS front-end with synchronized single-lead ECG acquisition was developed, as shown in **Figure 11**. The system employed direct-digital-synthesis excitation from 1 kHz to 1 MHz, three tetrapolar transthoracic measurement paths, lead-off detection, and channel-wise validation, thereby enabling synchronized acquisition of frequency-resolved bioimpedance and cardiac electrical activity through a shared wearable architecture, rather than through separate measurements for later comparison (Amin et al., 2026). Similarly, other platforms integrating bioimpedance with ECG, PPG, respiratory, or inertial signals have been investigated for cardiopulmonary monitoring, continuous physiological sensing, and activity recognition (Sel et al., 2020; Imtiaz et al., 2017; Hedayatipour and McFarlane, 2020). Collectively, these studies indicate that multimodal human bioimpedance systems are progressing from the simple addition of sensing channels toward coordinated cross-modal measurement within unified acquisition architectures.

Despite these advances, current multimodal human bioimpedance studies provide stronger evidence for the feasibility of sensor integration than for reliable exercise-state recognition. Most systems have been evaluated under resting, clinical, or controlled activity conditions, whereas prolonged or high-intensity exercise introduces continuous changes in posture, tissue geometry, sweating, and electrode contact. These changes not only reduce signal stability but also complicate the physiological correspondence among different modalities. ECG reflects cardiac electrical excitation over a short time scale, inertial signals closely follow mechanical movement, whereas impedance responses associated with respiration, perfusion, tissue deformation, and fluid redistribution may evolve more gradually. Simultaneous acquisition therefore does not necessarily ensure that different signals represent the same physiological event. Timestamp correction, resampling, and event-based alignment using cardiac, respiratory, or movement landmarks can improve temporal consistency (Wang et al., 2023; Xiao et al., 2022), but fusion models must still distinguish technical misalignment from genuine physiological latency. Exercise further changes both tissue current pathways and the electrode–skin interface, causing motion-related disturbances to overlap with meaningful impedance responses. Inertial references, adaptive correction, and signal-quality assessment can help identify unreliable signal segments (An et al., 2022; Moeyersons et al., 2021). However, movementassociated impedance variation should not be removed indiscriminately because muscle deformation, respiratory motion, and fluid redistribution are themselves physiologically relevant.

The reliability of multimodal interpretation also depends on whether the impedance channel preserves accurate frequency-dependent information. In BIS, current-source instability, front-end errors, leakage, and parasitic capacitance may distort high-frequency *R*(*f*) and *X*_c_(*f*) estimates, thereby biasing fitted Cole-model parameters and weakening physiological interpretation. This limitation was addressed by estimating high-frequency Cole-model characteristics without directly relying on potentially unreliable high-frequency resistance and reactance measurements (Razaghi et al., 2023). Tetrapolar sensing can reduce the influence of electrode–skin impedance, while lead-off detection and channel-wise validation help identify electrode-path failure and inconsistent measurements. Nevertheless, these measures cannot replace calibration and frequency-dependent system validation under realistic exercise conditions. Directly measured impedance samples and fitted BIS parameters should also be reported separately. Data fusion introduces an additional trade-off. Feature-level fusion can preserve relationships among cardiovascular, respiratory, tissue-electrical, and movement responses but remains sensitive to residual alignment errors and unreliable channels, whereas decision-level fusion may be more robust when one modality becomes temporarily unavailable. Future studies should evaluate not only overall recognition performance, but also the physiological correspondence, reliability, and independent contribution of each modality across exercise tasks and monitoring durations. At present, multimodal human bioimpedance is better regarded as a complementary framework than as a fully validated autonomous system for exercise-state assessment.

## 6 Conclusion and outlook

The reviewed evidence indicates different levels of methodological maturity across sports-health applications. BIA-based body-composition assessment and standardized hydration monitoring are comparatively established, whereas fatigue-related interpretation, EIM-based functional assessment, and multimodal exercise-state recognition require stronger validation. Throughout this review, human bioimpedance refers to the general measurement domain, whereas BIA, BIS, BIVA, and EIM represent distinct analytical or regional frameworks. Resistance and reactance are components of complex impedance, while impedance magnitude and PhA are derived from their relationship. Maintaining these distinctions prevents raw measurements, model outputs, and physiological interpretations from being treated as equivalent. Translation from laboratory or clinical settings to dynamic exercise nevertheless remains challenging. Sweating alters electrode–skin impedance, contraction changes tissue geometry and current pathways, and postural transitions, sensor displacement, and mechanical loading produce baseline shifts or transient artifacts. Because these disturbances can follow exercise rhythm and intensity, they may overlap with meaningful responses and cannot always be addressed using filters developed for stationary measurements.

Future research should prioritize acquisition protocols, processing methods, and validation frameworks tailored to dynamic exercise. Standardized reporting should include excitation frequency or frequency range, current amplitude, electrode configuration, anatomical current path, posture, measurement timing, and the convention used for reactance and PhA. Raw variables such as resistance, reactance, and impedance magnitude should be distinguished from BIA-derived estimates, BIS-fitted parameters, BIVA indices, and EIM outcomes. Wearable human bioimpedance systems also require stable electrode–skin interfaces, calibration across the operating frequency range, real-time signal-quality assessment, and synchronization with motion sensors. Motion-robust processing may reduce contraction-related fluctuations and movementinduced baseline shifts, while task-related impedance components should be retained when their origin can be verified against concurrent respiratory, mechanical, or physiological references. Reliable interpretation further requires sport-specific reference data, repeated-measurement calibration, and cross-validation against established physiological and functional benchmarks. Ultimately, longitudinal studies across diverse sporting populations, training regimens, and recovery conditions are needed to establish the physiological validity and practical utility of human bioimpedance-based monitoring.
